# Comparison of the safety and efficiency of temporary cardiac pacing methods during left bundle branch pacemaker implantation: Femoral vein pacing versus atrial spiral pacing with electrodes placed at the ventricle

**DOI:** 10.1002/clc.23992

**Published:** 2023-02-16

**Authors:** Zhenwei Li, Qingqing Xu, Ning Huangfu, Zewei Sun, Jia Su

**Affiliations:** ^1^ Department of Cardiology Ningbo Hospital of Zhejiang University Ningbo China; ^2^ Department of Nephrology Ningbo Hospital of Zhejiang University Ningbo China; ^3^ Department of Cardiology, The First Affiliated Hospital Zhejiang University School of Medicine Hangzhou China

**Keywords:** atrial spiral pacemaker electrode, femoral vein pacemaker electrode, left bundle branch pacemaker

## Abstract

**Background:**

Left bundle branch pacemakers (LBBPs) can better maintain ventricular electrical synchronization than traditional right ventricular pacing (RVP). Temporary cardiac pacing (TCP) is needed to ensure the safety of the operation in patients undergoing LBBP. Currently, there are two methods of installing TCP in conventional permanent pacemaker implantation. **Hypothesis**: To evaluate the safety and efficiency of replacing femoral vein pacing with atrial spiral pacing in the right ventricle for temporary cardiac pacing (TCP) during left bundle branch pacemaker (LBBP) implantation.

**Method:**

A total of 179 patients who underwent TCP during LBBP were selected for retrospective analysis from April 2019 to 2021 and divided into two groups: the atrial spiral electrode group (*n* = 76) and the femoral vein electrode group (*n* = 103). The following were observed: operation time; radiation dose; radiation time; operation expenses; hospitalization time; pacemaker parameters immediately after the operation and at 1 week, 1 month, 3 months, and 6 months after the operation; operation complications and femoral vein puncture point complications were observed in the two groups.

**Results:**

Compared to the femoral vein electrode group, the atrial electrode group had significantly lower operation times ([116.86 ± 24.63] versus [128.94 ± 25.27] min, *p* < 0.05), radiation doses ([805.07 ± 132.94] versus [846.42 ± 87.37] mgy, *p* < 0.05), and decreased risk of a displaced or dislodged temporary pacing electrode during the operation ([0.00%] versus [4.85%], *p* < 0.05). The atrial electrode group did not have significant operation costs or material costs associated with femoral vein temporary pacing electrode implantation. In addition, the atrial electrode group did not have an increased risk of pacemaker‐related infections, and the parameters of the pacemaker were unaffected. However, some puncture point complications appeared in the femoral vein electrode group (8 cases of local subcutaneous hematoma, 3 cases of pseudoaneurysms, 3 cases of arteriovenous fistula).

**Conclusion:**

The replacement of the femoral vein pacing electrode with an atrial spiral pacing electrode in the right ventricle for TCP during LBBP implantation was safe and effective.

## Graphical abstract

Figure 1: Femoral vein temporary pacing electrode application diagram. After femoral vein puncture, temporary cardiac pacing (TCP) electrodes were placed into the right ventricular apex through the inferior vena cava, right atrium, and tricuspid valve. Then, the end of the electrode was connected to the program‐controlled instrument to test the parameters satisfiedly for TCP.

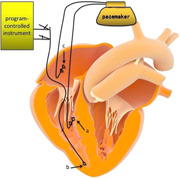

## 1 INTRODUCTION

1.1

Cardiac pacemaker implantation is the most common clinical treatment for slow arrhythmia.^1^ Left bundle branch pacemakers (LBBPs) can better maintain ventricular electrical synchronization than traditional right ventricular pacing (RVP). LBBPs can better improve cardiac structure and function and reduce the incidence of heart failure (HF) in patients with a high proportion of ventricular pacing, including high‐degree atrioventricular block (AVB) and atrial fibrillation (AF) with a slow ventricular rate.^2^ In addition, LBBP performed in patients with HF (low left ventricular ejection fraction [LVEF]) and complete left bundle branch block (LBBB) with the indication of traditional cardiac resynchronization therapy can correct ventricular conduction and improve cardiac function. Its feasibility and clinical efficacy have been confirmed (8−14). However, finding the pacemaker site and the process of screwing or fixing pacing electrodes can cause damage to the atrioventricular conduction system, which will cause syncope and cardiac arrest. Therefore, temporary cardiac pacing (TCP) is needed to ensure the safety of the operation in patients undergoing LBBP.^3^ Currently, there are two methods of installing TCP in conventional permanent pacemaker implantation: (1) After femoral vein puncture, TCP electrodes are placed into the ministry of the right ventricular apex through the inferior vena cava, right atrium, and tricuspid valve. Then, the end of the electrode is connected to the program‐controlled instrument for TCP. (2) The atrial spiral pacing electrode is placed into the right ventricle for TCP as a transition. After successfully placing the ventricular LBBP electrode, the transition of the spiral pacing electrode is pulled out and then implanted into the corresponding atrial parts. The purpose of the study was to compare the two methods in terms of operation time, radiation dose, radiation time, operation expenses, hospitalization time, complications (e.g., pacing electrode dislodgement or displacement, pneumothorax, hemothorax, cardiac perforation, pericardial tamponade, pacemaker pouch hematoma, pacemaker‐related infection), pacemaker parameters, and femoral vein puncture point complications (e.g., local subcutaneous hematoma, retroperitoneal hematoma, mediastinal hematoma, arteriovenous fistula, and false aneurysm).

## 2 DATA AND METHODS

### 2.1 Data

Inclusion criteria: Patients from 50 to 80 years of age with a diagnosis of HF with complete LBBB, paroxysmal AF with slow ventricular rate, and high‐degree AVB were retrospectively randomly selected from April 2019 to 2021 in the cardiac catheterization room in Ningbo Hospital of Zhejiang University and The First Affiliated Hospital of Zhejiang University Medical School. Patients who underwent TCP during LBBP was divided into an atrial electrode group and femoral vein electrode group. Five surgeons from the two centers who performed, on average, 100 independent pacemaker implantations, performed the TCP and LBBP procedures.

The exclusion criteria were as follows: allergy to contrast agents, acute HF, cardiac shock, pulmonary edema, electrolyte disorder, fever and infectious diseases, malignant tumor, acute myocarditis, autoimmune disease, blood coagulation disorders, and severe liver and kidney dysfunction.

### 2.2 Method

All methods were carried out in accordance with relevant guidelines and all experimental protocols were approved by by Ningbo Hospital of Zhejiang University (2022RS001). All patients or their family members were informed of the risks associated with TCP and LBBP and signed informed consent forms to undergo the procedures.

#### 2.2.1 Preoperative preparation

All patients discontinued aspirin, clopidogrel, warfarin, and traditional Chinese medicine 1 week before pacemaker implantation. First‐generation cephalosporin antibiotics were administered to prevent infection 24 h preoperatively and postoperatively. Routine blood counts, electrolyte levels, liver and kidney function tests, blood coagulation function indices, electrocardiograms, echocardiograms, chest computed tomography, and abdominal ultrasounds were examined before the operation.

#### 2.2.2 Pacing electrode and pacemaker

atrial active electrode: 5076‐52 (Medtronic), sheath pipe: C315 (Medtronic), ventricular LBBP electrode: 3830 (Medtronic), femoral vein temporary pacemaker electrode: PACEL dominate (St. Jude), the permanent pacemaker models: A3DR01 (Medtronic), SEDRL1 (Medtronic), and RED01 (Medtronic).

#### 2.2.3 TCP application

##### Femoral vein temporary pacing electrode implantation

**Figure 1:**
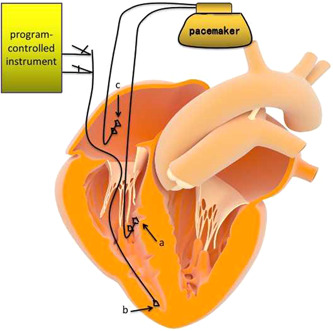
Femoral vein temporary pacing electrode application diagram. a, Ventricular spiral pacing electrode. b, Femoral vein temporary pacing electrode. c, Atrial spiral pacing electrode.

After femoral vein puncture, TCP electrodes were placed into the right ventricular apex through the inferior vena cava, right atrium, and tricuspid valve. Then, the end of the electrode was connected to the program‐controlled instrument to test the parameters satisfiedly for TCP (as shown in Figure 1). The TCP protective application frequency was set to 40/min. Once the LBBP was implanted, the temporary pacing electrode was pulled out. Then, the puncture point was fixed, compressed, and bandaged.

##### Atrial spiral pacing electrode implantation

**Figure 2:**
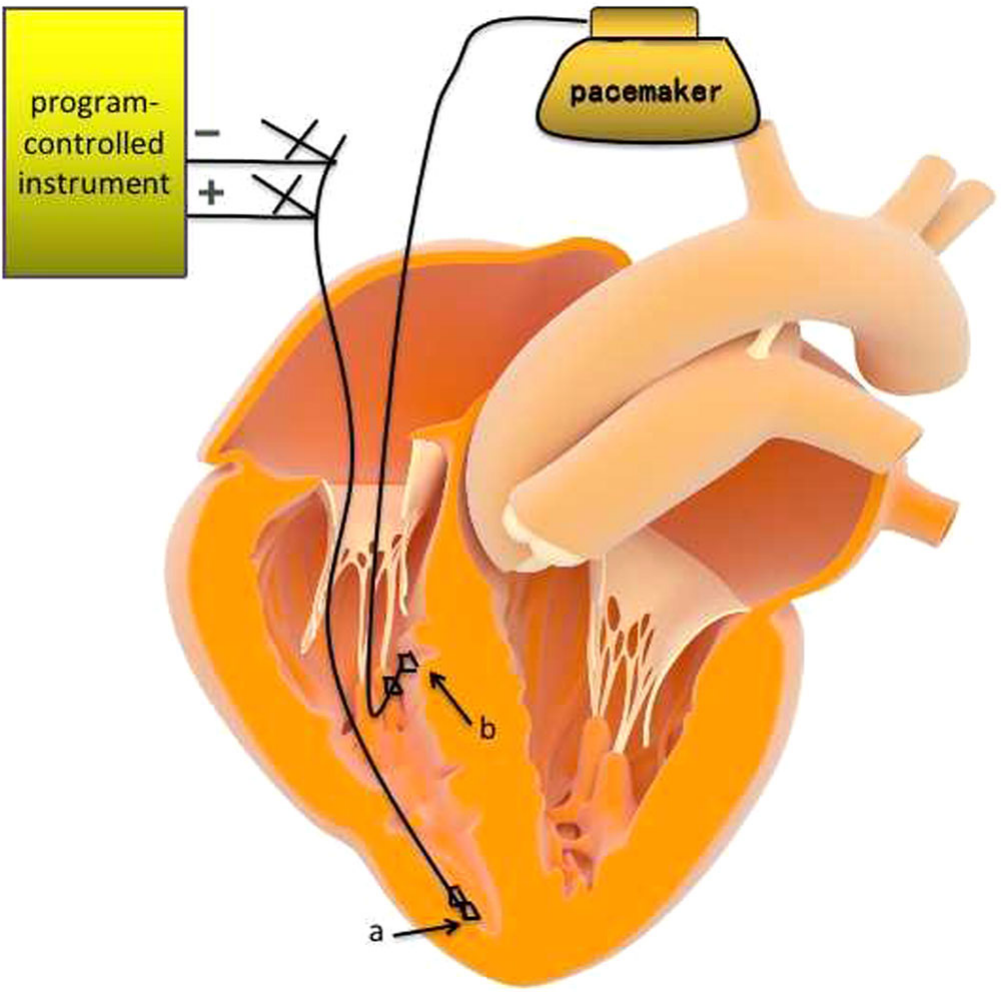
Atrial spiral pacing electrode application diagram. a, An atrial spiral pacing electrode was implanted at the right ventricular apex for temporary pacing. After the right ventricular pacing electrode was placed properly, atrial electrodes were pulled out and implanted at the corresponding atrial location. b, Ventricular spiral pacing electrode.

An atrial spiral pacing electrode was implanted properly at the right ventricular apex, the another end of the electrode was connected to the program‐controlled instrument to test the parameters satisfiedly. Then the atrial electrode was spiraled into the myocardium by the fixation tool for temporary pacing as a transition. The TCP protective application frequency was set to 40/min. After the RVP electrode was implanted properly, the parameters were tested and satisfied, connecting to the pacemaker ventricular electrode hole. Then, the transition of atrial electrodes was spiraled out by the fixation tool and implanted at the corresponding atrial location, which was connected to the pacemaker atrial electrode hole (as shown in Figure 2).

#### 2.2.4 LBBP implantation

A 3−5 cm incision was made left of the subclavian vein, and a delivery catheter and guidewire (C315 sheath pipe; Medtronic) were inserted through the subclavian vein or axillary vein to a place near the tricuspid valve opening. Then, the ventricular lead (3830; Medtronic) was fed through the C315 sheath pipe. The electrode found the suitable position as a target reference of the tricuspid ring in the right front 30° oblique position under exposure. Then, the electrode distal end stuck out of the sheath pipe near the interval of the tricuspid valve with 2 V pacing. When the incisures appeared in the rising QS wave on the V1 lead, electrode screwing was performed at this site. Once the left bundle branch proved to be capturing the pacing and adjusting parameters satisfactorily, the electrode was fixed clockwise and then placed at the corresponding right atrium location, connected to the pacemaker after adjustment parameters, closing the wound in the end.

### 2.3 Research aims and parameters

The primary endpoints of this study were as follows: operation time, radiation dose, radiation time, operation expenses, hospitalization time, complications (temporary pacing electrode dislodgement or displacement, permanent pacing electrode dislodgement or displacement, pneumothorax, hemothorax, cardiac perforation, pericardial tamponade, pacemaker pouch hematoma, pacemaker infection), and femoral vein puncture point complications (local hematoma, retroperitoneal hematoma, arteriovenous fistula, and pseudoaneurysms).

### 2.4 Statistical analysis

SPSS version 22.0 (IBM) was used to statistically analyze the data, and continuous variables are expressed as the mean ± SD. Independent sample *t*‐tests were used for comparisons between groups. Classification variables were expressed by frequency or percentage using the *χ* ^2^ test or Fisher's exact test. *p* < 0.05 indicates that the difference was statistically significant.

## 3 RESULTS

### 3.1 Basic data

One hundred seventy‐nine patients were enrolled; of them, 98 patients were male, 82 patients were female, the mean age was 68.66 ± 12.297 years old, 84 patients had hypertension, 66 patients had hyperlipidemia, 67 patients had a history of diabetes, 16 patients had a history of stroke, and 16 patients had coronary heart disease. There was no significant difference between the two groups in age, sex, body mass index, medical history, pacemaker indications, pacemaker model, heart ejection fraction, creatinine, hemoglobin, or absorbable line used (*p* > 0.05). The patient base data for the two groups are shown in Table 1.

**Table 1:** Patients' base data in both groups.

Item	Atrial spiral electrode (76 cases)		Femoral vein pacing electrode (103 cases)		*p* Value
Age (years)	68.38 ± 12.20		69.04 ± 12.49		0.72
Sex (male)	61.17%		59.21%		0.79
BMI (kg/m^2^)	22.70 ± 4.76		23.1 ± 5.04		0.57
Medical history (case)					
Stroke	8 (7.89%)		8 (7.77％)		0.98
CHD	7 (9.21%)		9 (8.74%)		0.91
Diabetes	23 (30.26%)		24 (25.24%)		0.17
Hypertension	33 (44.42%)		51 (49.51%)		0.42
Hyperlipidemia	24 (31.58%)		42 (40.78%)		0.21
Pacemaker indications (case)					0.56
Heart failure with LBBB	12 (15.79%)		13 (12.62%)		
High‐degree AVB	56 (73.68%)		81 (78.64%)		
Paroxysmal AF with slow rate	8 (10.53%)		9 (8.74%)		
Pacemaker type (case)					0.60
RED01	57 (75.00%)		75 (72.81%)		
A3DR01	11 (14.47%)		20 (19.41%)		
SEDRL1	8 (10.53%)		8 (7.76%)		
Heart ejection fraction (%)	51.32 ± 8.53		51.07 ± 8.70		0.85
Hemoglobin (g/L)	135.03 ± 9.13		133.78 ± 9.17		0.37
Creatinine (umol/L)	87.34 ± 22.16		88.54 ± 20.58		0.71
Absorbable line used (case)	37 (48.68%)		45 (43.69%)		0.46

Abbreviations: AF, atrial fibrillation; AVB, atrioventricular block; BMI, body mass index; CHD, coronary heart disease; LBBB, left bundle branch block.

### 3.2 The procedural characteristics compared in both groups

Both groups of patients were successfully implanted with pacing electrodes and pacemakers. The atrial electrode group had an operation time of 116.86 ± 24.63 min, which was less than that of the femoral vein electrode group (128.94 ± 25.27 min), and the difference was statistically significant (*p* < 0.05). TCP was initiated in 33 (43.42%) patients in the atrial spiral electrode group. Meanwhile, TCP was initiated in 42 (40.78%) patients in the femoral vein pacing electrode group. In the atrial electrode group, the X‐ray exposure dose (805.07 ± 132.94) mgy was lower than that in the femoral vein (846.42 ± 87.37) mgy electrode group, and the difference was statistically significant (*p* < 0.05). Regarding the operation cost, the atrial electrode group did not undergo femoral vein temporary pacing electrode implantation surgery, and surgical consumables cost 2200 Yuan (RMB). The X‐ray exposure time in the two groups was not significantly different (14.76 ± 4.63) versus (15.30 ± 3.29) min (*p* > 0.05). Regarding surgical complications, there was no temporary pacing electrode dislodgement in the atrial electrode group, while the femoral vein electrode groups had 5 cases of electrode dislodgement, accounting for 4.85%, and the difference was statistically significant (*p* < 0.05). Regarding other complications, the two groups had no permanent pacing electrode dislodgement. The atrial electrode group had no pneumothorax, but 2 cases of pneumothorax occurred in the femoral vein electrode group, accounting for 1.94%, and there was no significant difference (*p* > 0.05). Hemothorax did not occur in the atrial electrode group, but 2 cases occurred in the femoral vein electrode group (1.94%), and there was no significant difference (*p* > 0.05). There were no cardiac perforations or pericardial tamponades in the two groups. The atrial electrode group had 3 cases of pacemaker pouch hematoma, accounting for 3.95%, and 2 cases of pacemaker pouch hematoma occurred in the femoral vein electrode group, accounting for 1.94%; there was no significant difference (*p* > 0.05). Eight cases of pacemaker‐related infection occurred in the atrial electrode group, accounting for 10.53%, and 12 cases occurred in the femoral vein electrode group, accounting for 11.65%; there was no significant difference (*p* > 0.05). Regarding complications at the femoral vein puncture point, the femoral vein electrode group had an ecchymoma in 8 cases (7.77%), arteriovenous fistula in 3 cases (2.91%), pseudoaneurysms in 3 cases (2.91%), and no retroperitoneal hematoma; moreover, there were no puncture complications in the atrial electrode group. There were no significant differences in hospitalization time between the two groups (7.27 ± 1.72 vs. 7.39 ± 1.68 days) (*p* > 0.05). The procedural characteristics in the two groups are shown in Table 2. In terms of pacemaker parameters, both groups of parameters were satisfactory immediately after surgery and 1 week, 1 month, 3 months, and 6 months after discharge, and there was no statistically significant difference in parameters between the two groups. The pacemaker parameters are shown in Table 3.

**Table 2:** The procedural characteristics compared in both groups.

Item	Atrial spiral electrode (76 cases)	Femoral vein pacing electrode (103 cases)	*p* Value
Operation time (min)	116.86 ± 24.63	128.94 ± 25.27	0.002
TCP initiation ratio	33 (43.42%)	42 (40.78%）	0.18
X‐ray exposure time (min)	14.76 ± 4.63	15.30 ± 3.29	0.37
X‐ray exposure dose (mgy)	805.07 ± 132.94	846.42 ± 87.37	0.013
Temporary electrode implantation surgery consumables cost (Yuan)	0 ± 0.00	2200 ± 0.00	NA
Surgical complications (case)			
Temporary electrode dislodgement	0 (0.00%)	5 (4.85%)	0.02
Permanent electrode dislodgement	0 (0.00%)	0 (0.00%)	1.00
Pneumothorax	0 (0.00%)	2 (1.94%)	0.23
Hemothorax	0 (0.00%)	2 (1.94%)	0.23
Cardiac perforation, pericardial tamponade	0 (0.00%)	0 (0.00%)	1.00
Pacemaker pouch hematoma	3 (3.95%)	2 (1.94%)	0.41
Pacemaker related infection	8 (10.53%)	12 (11.65%)	0.81
Complications in femoral vein puncture point (case)			
Ecchymoma	0 (0.00%)	8 (7.77%)	0.01
Retroperitoneal hematoma	0 (0.00%)	0 (0.00%)	0.39
Pseudoaneurysms	0 (0.00%)	3 (2.91%)	0.13
Arteriovenous fistula	0 (0.00%)	3 (2.91%)	0.13
Hospitalization time (day)	7.27 ± 1.72	7.39 ± 1.68	0.64

Abbreviation: TCP, temporary cardiac pacing.

**Table 3:** The pacemaker parameters compared in both groups.

Item	Atrial spiral electrode (76 cases)	Femoral vein pacing electrode (103 cases)	*p* Value
Pacemaker parameters immediately after surgery			
Atrial threshold (V)	1.04 ± 0.28	1.02 ± 0.27	0.61
Atrial impedance (Ω)	588.72 ± 68.85	595.82 ± 69.06	0.50
Ventricular threshold (V)	1.01 ± 0.11	0.99 ± 0.12	0.42
Ventricular impedance (Ω)	651.14 ± 102.85	632.49 ± 88.88	0.20
Pacemaker parameters 1 week after discharge			
Atrial threshold (V)	1.14 ± 0.18	1.11 ± 0.13	0.20
Atrial impedance (Ω)	592.64 ± 65.60	587.20 ± 72.79	0.73
Ventricular threshold (V)	0.86 ± 0.11	0.86 ± 0.10	0.42
Ventricular impedance (Ω)	540.53 ± 137.17	583.76 ± 153.27	0.06
Pacemaker parameters 1 month after discharge			
Atrial threshold (V)	1.13 ± 0.19	1.11 ± 0.15	0.32
Atrial impedance (Ω)	585.54 ± 71.23	570.92 ± 69.47	0.13
Ventricular threshold (V)	0.77 ± 0.21	0.74 ± 0.19	0.40
Ventricular impedance (Ω)	538.55 ± 135.68	575.89 ± 158.31	0.10
Pacemaker parameters 3 month after discharge			
Atrial threshold (V)	1.13 ± 0.20	1.14 ± 0.22	0.77
Atrial impedance (Ω)	551.05 ± 148.17	592.44 ± 161.38	0.08
Ventricular threshold (V)	0.75 ± 0.25	0.75 ± 0.22	0.97
Ventricular impedance (Ω)	537.21 ± 139.37	573.08 ± 156.84	0.12
Pacemaker parameters 6 month after discharge			
Atrial threshold (V)	1.15 ± 0.32	1.14 ± 0.28	0.87
Atrial impedance (Ω)	575.13 ± 135.13	613.65 ± 143.65	0.07
Ventricular threshold (V)	0.81 ± 0.32	0.77 ± 0.29	0.40
Ventricular impedance (Ω)	555.00 ± 158.22	570.65 ± 152.56	0.51

## 4 DISCUSSION

LBBP was first proposed in 2017. Huang et al.^4^ screwed the pacing electrode at the distal end of the bundle to the left side of the septum in a patient with HF with complete LBBB and captured the left bundle branch. The pacing threshold was as low as 0.5 V/0.5 ms. After the 1‐year follow‐up, LVEF was improved (32%−62%), and the pacing threshold was stable. Subsequent studies showed that LBBP could better maintain left and right ventricular electrical synchronization, shorten the QRS time limit, and improve cardiac structure and function in patients who require a high proportion of ventricular pacing compared with traditional RVP, thereby reducing the incidence of HF.^2^, ^3^, ^5^, ^6^, ^7^, ^8^ Subsequently, LBBP in patients with existing HF (decreased LVEF) with complete LBBB could correct ventricular conduction and improve cardiac function.^9^, ^10^, ^11^, ^12^, ^13^, ^14^, ^15^, ^16^ The results of the first multicenter prospective study in 2020 showed that when patients with nonischemic cardiomyopathy with LVEF <50% and LBBB underwent LBBP, the QRS time limit decreased compared with the baseline, and the LVEF of 75% of the patients increased significantly by more than 50% by the 1‐year follow‐up, which further confirmed that LBBP could restore the electrical synchronization of LBBB patients and improve the cardiac structure and function of patients with HF.^15^ For patients with HF complicated with LBBB, atrial activation can only be transmitted to the ventricle through the right bundle branch with relatively normal or simultaneous conduction lesions. Even if right bundle branch conduction is normal, right bundle branch conduction block often occurs due to mechanical pressure contact during the operation of the electrode on both the atrial and ventricular sides of the tricuspid septal valve. In patients with possible lesions of the right bundle branch, mechanical damage to the already fragile right bundle branch conduction and the occurrence of high AVB will often be caused during the left bundle branch search for suitable pacing sites or during the screwing in and fixation of the pacing leads. For patients with slow ventricular rate AF or high‐degree atrioventricular conduction block, in the process of finding LBBP sites or fixing 3830 electrodes, the previously diseased atrioventricular conduction system could cause further damage and further reduce the heart rate, resulting in cardiac arrest, syncope, and other events. Therefore, TCP is required during LBBP to ensure the safety of the operation.^3^, ^16^

In this study, we selected ideal candidates for LBBP.^17^, ^18^, ^19^, ^20^ Selection included patients with HF with a complete LBBB and patients who require a high proportion of ventricular pacing, such as paroxysmal AF with a slow ventricular rate and patients with a high‐degree atrioventricular transmission block. Two conventional methods of temporary pacing during permanent pacemaker operation were compared. Our study result was similar to that of previous literature,^21^ which was carried out in a His‐bundle pacing operation. In this study, we found that TCP was initiated in more than 40% of patients during LBBP. Protective temporary pacing protection should be implemented in patients with LBBB, intermittent high AVB, high AVB with syncope history, or AF with slow ventricular rate. Compared with the femoral vein temporary pacing electrode group, the atrial electrode group had a significantly reduced operation time, which may be because the femoral vein electrode group required more time for disinfection, towel laying, puncture, and sheath placement. The atrial electrode group required less radiation because the femoral vein electrode group required more radiation on the path from the femoral vein to the right atrium. In this study, there were 5 patients with femoral vein temporary pacing electrode dislodgement during permanent pacing implantation, including 2 patients with Adams−Stokes syndrome. However, there was no atrial spiral electrode dislodgment or displacement in this study. A higher risk of electrode displacement or dislodgement during femoral vein temporary pacing. This phenomenon is consistent with previous literature research.^22^, ^23^ This may be due to the end of the traditional femoral vein temporary pacing electrode being cylindrical, with a smooth surface that is difficult to affix and easy to dislodge. Meanwhile, the atrial spiral pacing electrode group used a soft lead with a spiral electrode tip affixed at the right ventricular apex to obtain a stable pacing threshold and electrode impedance, which reduced the risk of electrode dislodgement. In addition, the patients were uncomfortable during the operation, and involuntary movement of the legs easily dislodged the electrode. The patients urgently required adjustment of the electrode position after temporary pacing electrode slippage. Adjustment resulted in pacing rhythm restoration, and the 2 patients regained consciousness. These patients' surgical safety risk and surgical infection risk also increased throughout the process. Additionally, the atrial electrode group did not produce puncture point complications as occurred in the femoral vein temporary pacing group, reducing operation pain in patients. This reduced the overall economic burden of operation cost from pain medication use as well as disposable equipment use due to femoral vein pacing electrode implantation. Finally, in this study, we also found that the atrial spiral electrode group had lower infection rates related to permanent pacemaker implantation. The pacing parameters of the pacemakers were not affected during postoperative follow‐up.

In conclusion, it is safe and feasible to use an atrial spiral electrode placed at the ventricle instead of a femoral vein pacing electrode for temporary pacing during LBBP implantation. It reduces the operation time, radiation dose, risk of temporary pacing electrode displacement or dislodgment, risk of puncture point complications, and overall operation expenses from disposable equipment due to lead displacement. It will not increase the pacemaker‐related infection rate and will not affect the pacing parameters of the pacemaker. The limitations of this study are as follows: (1) For patients with sick sinus syndrome with normal atrioventricular conduction, atrial sensing, and pacing are completely in line with physiology and do not need LBBP. For patients with permanent AF, only single chamber pacing is needed, and atrial electrode implantation is not needed. Therefore, these two types of patients were not included in this study, so there may be selection bias. (2) This study is a retrospective study; thus, the conclusion may have selection bias. Finally, this study is a two‐center clinical study, the included sample size was large, and the follow‐up time was long. More cases are needed to further verify the conclusion.

## AUTHOR CONTRIBUTIONS

Zhenwei Li, Jia Su, Qingqing Xu, and Ning Huangfu wrote the main manus cript text. Zewei Sun prepared Figures 1, 2 and Tables 1, 2, 3. All authors reviewed the manuscript.

## CONFLICT OF INTEREST STATEMENT

The authors declare no conflict of interest.

## Data Availability

The data sets used and/or analyzed during the current study available from the corresponding author on reasonable request.
